# Distress screening and management for adolescents and young adults: 2022 NCI community oncology program landscape assessment

**DOI:** 10.1007/s00520-026-11009-x

**Published:** 2026-07-18

**Authors:** Karly M. Ingram, Lingyun Ji, Han-Wei V. Wu, Kristin M. Bingen, Emily V. Dressler, Carol Kittel, Jordan Gilleland Marchak, Meghan E. McGrady, Chandylen L. Nightingale, Alexandra M. Psihogios, Joanna M. Robles, Michael Roth, Susan K. Parsons, Melissa P. Beauchemin

**Affiliations:** 1 Department of Psychology, East Carolina University, 1000 E. 5th St., MS 565, Greenville, NC USA; 2 Department of Population and Public Health Sciences, Keck School of Medicine, University of Southern California, Los Angeles, USA; 3 Department of Pediatrics, Memorial Sloan Kettering Cancer Center, New York, USA; 4 Department of Pediatrics, Medical College of Wisconsin, Milwaukee, USA; 5 Department of Biostatistics and Data Science, Wake Forest University School of Medicine, Winston-Salem, USA; 6 Department of Pediatrics, Aflac Cancer & Blood Disorders Center, Emory University School of Medicine, Atlanta, USA; 7 Division of Behavioral Medicine and Clinical Psychology, Cincinnati Children’s Hospital Medical Center, Cincinnati, USA; 8 Department of Pediatrics, University of Cincinnati College of Medicine, Cincinnati, USA; 9 Department of Social Sciences and Health Policy, Wake Forest University School of Medicine, Winston-Salem, USA; 10 Department of Medical Social Sciences, Northwestern University Feinberg School of Medicine, Chicago, USA; 11 Department of Pediatrics – Hematology and Oncology, Wake Forest University School of Medicine, Winston-Salem, USA; 12 Department of Pediatrics, MD Anderson Cancer Center, Houston, USA; 13 Institute for Clinical Research and Health Policy Studies and Division of Hematology/Oncology, Tufts Medical Center, Boston, USA; 14 School of Nursing, Columbia University, New York, USA

**Keywords:** Adolescent and young adult, Distress screening, Distress management, Mental health, Access to care

## Abstract

**Purpose:**

Adolescent and young adult cancer patients (AYAs, aged 15–39) are at an elevated risk of experiencing psychosocial distress. Furthermore, most AYAs receive care in community oncology settings where little is known about distress screening and management for AYAs. We examined distress screening at National Cancer Institute Community Oncology Research Program (NCORP) across 100 practices previously identified as AYA-treating.

**Methods:**

Using data from the 2022 Landscape Assessment survey, distress screening and availability of mental health care were evaluated. Univariable and multivariable analyses examined associations between on-site availability of mental health services and AYA-relevant practice characteristics (e.g., Children’s Oncology Group affiliation, number of new AYAs annually, AYA program).

**Results:**

Nearly all (91%) AYA-treating practices reported routinely screening for distress, with 47% using the National Comprehensive Cancer Network (NCCN) Distress Thermometer. Additionally, 23% of practices reported they do not have mental health services onsite. Practices treating ≥ 100 new AYAs annually were more than 3 times as likely to have onsite mental health services than practices treating < 100 new AYAs annually in multivariable models (OR = 3.09, 95% CI = 1.04, 9.18; *p* = 0.042).

**Conclusions:**

Although distress screening was widely conducted in AYA-treating NCORP practices, findings raise concerns about the sensitivity and specificity of measures used and access to onsite cancer-specific psychosocial care. Ultimately, the goal of distress screening is referral for assessment and intervention; referral to off-site services may create access barriers. Future studies should focus on understanding whether those who experience distress can and do access cancer-specific psychosocial care, and barriers to doing so.

**Supplementary Information:**

The online version contains supplementary material available at 10.1007/s00520-026-11009-x.

## Introduction

As of 2020, there were more than 2.1 million survivors of adolescent and young adult cancer (AYAs, diagnosed between ages 15 to 39 years) living in the USA [1]. AYA cancer patients experience disruptions to normative life and delays in achieving developmental milestones, such as completing education, pursuing intimate relationships, and establishing independence. This often results in elevated levels of psychosocial distress, which is defined as “a multifactorial unpleasant experience of a psychological (ie, cognitive, behavioral, emotional), social, spiritual, and/or physical nature that may interfere with one's ability to cope effectively with cancer, its physical symptoms, and its treatment."” [2, 3]. Previous research suggests that AYA cancer patients experience greater levels of distress than either older or younger patients and their healthy peers [4, 5]. During the first year following a cancer diagnosis, 35% of AYAs report experiencing clinically significant distress at least once, rates that exceed population norms [6]. Since distress can negatively impact the quality of life and short and long-term health outcomes of these patients [7], screening for and addressing psychosocial distress in AYAs is an important component of providing comprehensive cancer care for this vulnerable population.

Multiple national cancer organizations have published guidelines recommending that adult patients with cancer, including AYAs, should be screened for distress at regular intervals during their cancer care, particularly during important transition points such as time of initial diagnosis, start of treatment, and transition to survivorship [2, 8–10]. For AYAs treated in the pediatric setting, routine assessment of psychosocial care needs, including distress screening, is an established standard of care [10, 11]. However, while the majority of AYAs with cancer are treated in community oncology settings [12], almost all of the research on this population has been conducted at large academic medical centers. Among community oncology centers that treat AYAs, it is unknown whether distress is being screened for, how it is being evaluated, and whether mental health care is available to AYAs who report elevated levels of distress. These knowledge gaps stall efforts to ensure that all AYAs can gain access to the psychosocial support they need.

The goal of the current study is to describe distress screening and management in community oncology practices that treat AYAs. As best practices for screening recommend that assessments are used to provide tailored intervention, we also explored associations between practice group characteristics and the availability of mental health services.

## Methods

This study used data collected via “The 2022 Landscape Assessment,” a collaborative effort representing all National Cancer Institute Community Oncology Research Program (NCORP) research bases, including the Children’s Oncology Group (COG) and led by the Wake Forest NCORP Research Base. The goal of NCORP is to bring cancer clinical trials and care delivery research to people in their communities. NCORP is comprised of both Minority/Underserved and non-Minority/Underserved community practices. Minority/Underserved practices are defined as academic centers at which at least 30% of the patient population is from underrepresented groups (e.g., rural, racial and ethnic minority groups) [13].

The detailed methodology for the Landscape Assessment survey has been previously described [14]; briefly, the survey aimed to describe infrastructure and current resources to understand the capacity to conduct cancer care delivery research at NCORP practice groups. Prior work established criteria for determining an AYA-treating NCORP practice in the context of this survey as having ≥ 5% of their annual cases between the ages of 15 and 39, treating ≥ 50 AYAs annually, and/or having a dedicated AYA program [15]. In the present study, we conducted all analyses using data from the 100 practices that met at least one of these criteria. Practices were further categorized as having COG affiliation, treating above or below the national average of patients insured by Medicaid or uninsured via comparison to 2023 Census Bureau data [16], and having a dedicated pediatric oncology program.

Exact questions and response options asked of practices are provided in Supplemental Table 1. Briefly, practices were asked whether they had a dedicated Pediatric or AYA Oncology program and, if so, what services this program provided from a list of response options, including an “other” option with a space to provide specifics. To assess distress screening, practices were asked whether they “routinely screen for distress (e.g., evaluate symptoms of anxiety and depression or psychosocial wellbeing in general) in your oncology patients”, and if so, what instruments are used and the strategies used to manage patients who screen positive for distress. Practices were also asked whether they used other Patient-Reported Outcomes (PROs) to inform clinical care, and if so, what instruments were used for this purpose. Finally, practices were asked about the availability of mental health care for oncology patients; if the practice indicated the availability of such services, they were asked to indicate what types of mental health care were available.

Practice groups were characterized using descriptive statistics with regards to Minority/Underserved classification, COG affiliation, availability of a dedicated pediatric or AYA program, provision of services to patients with Medicaid above the national average of 30%, types of practices included in the affiliate/subaffiliate, number of new AYAs treated annually, and ownership (e.g., independently owned, publicly owned, University-owned). Text responses provided to questions that allowed an “Other” option were recoded into existing categories if appropriate (e.g., the practice did not check the option for Patient Health Questionnaire (PHQ) but wrote in “PHQ-2” when specifying their “Other” response).

Outcomes of interest, i.e., psychosocial screening practices and availability of mental health care, were examined using descriptive statistics. Univariable and multivariable logistic regression models were used to examine associations between practice group characteristics (as described above) and onsite availability of mental health care (i.e., responding “Yes” to the item “Are mental health services available for oncology patients at your affiliate/subaffiliate?”). Variables that met a threshold value of *p* < 0.10 in univariable models were included in the multivariable models. Odds ratios were calculated for models in which all cells had a value of at least 1. All reported *p*-values are two-sided. Analyses were performed using STATA 18 [17].

## Results

### Characteristics of NCORP practices that treat AYAs

Of the 100 AYA-treating practices [15], 24% were COG practices; further, 64% did not have a Pediatric Oncology Program and 80% did not have an AYA Oncology Program. Practice affiliates most often included outpatient oncology clinics (84%), closely followed by inpatient services for oncology patients (81%). Most practices were large regional multi-state health systems that included a health insurance plan (54%). Additional characteristics are presented in Table 1.

**Table 1:** Characteristics of NCORP practices that treat AYAs (*N* = 100)

Characteristic	Practices	
	*n*	(%)
NCORP Practice Affiliation		
Minority/Underserved practice	25	(25%)
Non-Minority/Underserved practice	75	(75%)
Practice Settings^a^		
Outpatient oncology clinics in or on a hospital campus	84	(84%)
Free-standing outpatient oncology clinics or private/group practices	54	(54%)
Inpatient services for oncology patients	81	(81%)
Children’s hospital that treats pediatric oncology patients	32	(32%)
Affiliate/sub-affiliate Ownership		
Independently owned (i.e. single hospital, small regional network)	18	(18%)
Large regional multi-state health system that does include a health plan	54	(54%)
Large regional multi-state health system that does NOT include a health plan	12	(12%)
HMO/Payer owned	1	(1%)
Publicly owned	8	(8%)
University owned	4	(4%)
Other	3	(3%)
AYA Population Served		
< 50 new oncology cases ages 15–39 per year	22	(22%)
50–99 new oncology cases ages 15–39 per year	28	(28%)
100–199 new oncology cases ages 15–39 per year	22	(22%)
200–299 499 new oncology cases ages 15–39 per year	14	(14%)
> = 500 new oncology cases ages 15–39 per year	6	(6%)
# of new oncology cases ages 15–39 per year not reported	8	(8%)
AYA Oncology Program		
Yes	20	(20%)
No	80	(80%)
Pediatric Oncology Program		
Yes	36	(36%)
No/No Response	64	(64%)
NCORP practices serving patients above the national average (percentage) by insurance type^b^		
Medicaid	17	(17%)
Uninsured	12	(12%)

^a^Practices were allowed to choose multiple options so percentages sum to greater than 100

^b^The national average (percentage) of the population covered by Medicaid is 30%; the national average (percentage) of the population who are uninsured is 10%

### Psychosocial screening practices

Almost all (*n* = 91, 91%) practices surveyed reported routinely screening for “distress, symptoms of anxiety and depression, or psychosocial well-being in general.” The NCCN Distress Thermometer [18] was the most commonly reported tool used for this purpose (*n* = 49, 54%), with many practices also using the Patient Health Questionnaire (PHQ) (*n* = 41, 45%), although the specific number of items was not elicited. Other PROs were used by 34% of practices (*n* = 34) with heterogeneity noted in the symptom(s) assessed. Among these practices, the use of a single-item symptom severity rating was most common (*n* = 14, 41%). Additional details about the use of distress screening and PROs for clinical care are presented in Table 2.

**Table 2:** Psychosocial screening and mental health services available among NCORP practices that treat AYAs

	Total		Sites with Pediatric Oncology Program		Sites without Pediatric Oncology Program	
Routinely screens for distress in oncology patients	*n* = *100*		*n* = *36*		*n* = *64*	
	*n*	(%)	*n*	(%)	*n*	(%)
Yes	91	(91%)	33	(92%)	58	(91%)
No	9	(9%)	3	(8%)	6	(9%)
Instruments used for distress screening^a,b^	*n* = *91*		*n* = *33*		*n* = *58*	
	***n***	(%)	***n***	(%)	***n***	(%)
NCCN Distress Thermometer	49	(54%)	17	(52%)	32	(55%)
Patient Health Questionnaire (PHQ)	42	(46%)	15	(45%)	27	(47%)
Generalized Anxiety Disorder Scale (GAD-7)	22	(24%)	12	(36%)	10	(17%)
Patient-Reported Outcome Measurement Information System (PROMIS)	9	(9%)	3	(9%)	6	(10%)
Hospital Anxiety and Depression Scale (HADS)	5	(5%)	3	(9%)	2	(3%)
Edmonton Symptom Assessment System (ESAS)	4	(4%)	4	(12%)	0	(0%)
Psychosocial Assessment Tool (pediatric only)	4	(4%)	4	(12%)	0	(0%)
Other^c^	13	(14%)	5	(15%)	8	(14%)
Strategies used to manage patients who screen positive for distress^a,b^	*n* = *91*		*n* = *33*		*n* = *58*	
	***n***	(%)	***n***	(%)	***n***	(%)
Referral to an onsite service (e.g., counselor, mental health professional)	76	(84%)	28	(85%)	48	(83%)
Oncology provider assesses and manages patient	62	(68%)	20	(61%)	42	(72%)
Referral to an outside counseling service/mental health professional	53	(58%)	18	(55%)	35	(60%)
Referral to primary care provider	36	(40%)	9	(27%)	27	(47%)
Other^d^	3	(3%)	1	(3%)	2	(3%)
Routinely uses other patient reported outcomes (PROs) to inform clinical care for oncology patients (***n*** = 100)	*n* = *100*		*n* = *36*		*n* = *64*	
	***n***	(%)	***n***	(%)	***n***	(%)
Yes	34	(34%)	10	(28%)	24	(37%)
No	66	(66%)	26	(72%)	40	(63%)
PRO tools routinely used clinically^a,e^	*n* = *34*		*n* = *10*		*n* = *24*	
	***n***	(%)	***n***	(%)	***n***	(%)
Single-item symptom severity rating (e.g., pain)	14	(41%)	5	(50%)	9	(38%)
Patient-Reported Outcome Measurement Information System (PROMIS)	7	(21%)	3	(30%)	4	(17%)
FACT: Functional Assessment of Cancer Therapy	6	(18%)	0	(0%)	6	(25%)
ESAS: Edmonton Symptom Assessment Scale	4	(12%)	2	(20%)	2	(8%)
EORTC: European Organization for Research and Treatment of Cancer	2	(6%)	2	(20%)	0	(0%)
SF-36: Short Form Survey	2	(6%)	1	(10%)	1	(4%)
Other^f^	11	(32%)	3	(30%)	8	(33%)
Mental health services available for oncology patients	*n* = *100*		*n* = *36*		*n* = *64*	
	***n***	(%)	***n***	(%)	***n***	(%)
Yes	76	(76%)	32	(89%)	44	(69%)
No, but referral relationships with community mental health providers available	23	(23%)	4	(4%)	19	(30%)
No	1	(1%)	0	(0%)	1	(2%)
Specific mental health services offered^a,f^	*n* = *76*		*n* = *32*		*n* = *44*	
	***n***	(%)	***n***	(%)	***n***	(%)
Individual psychosocial or behavioral therapy	75	(99%)	32	(100%)	43	(98%)
Group psychosocial services	60	(79%)	26	(81%)	34	(77%)
Education classes around self-care for mental health	55	(72%)	22	(69%)	33	(75%)
Couples and family therapy	52	(68%)	21	(66%)	31	(70%)
Help in getting respite care	48	(63%)	20	(63%)	28	(63%)
Other^g^	6	(8%)	2	(6%)	4	(9%)

^a^Practices were allowed to choose multiple options so percentages sum to greater than 100

^b^Practices were only asked to respond to this item if they indicated that they routinely screen for distress in oncology patients

^c^Includes Columbia Suicide Scale (CSSR-C), Palliative Performance Scale, Peds-QOL, PrimeMD, SUDs, locally developed distress screening tools, and psychosocial assessment by clinical team members

^d^Includes “visit with Nurse Navigator or Social Worker”, “on site social worker screens for services,” and “referral to chaplain, dietitian, social worker, or financial navigator”

^e^Practices were only asked to respond to this item if they indicated that they routinely use other Patient Reported Outcomes (PROs) to inform clinical care for oncology patients

^f^Includes Electronic Symptom Management for Cancer Patients (ESyM), PHQ, GAD-7, Psychosocial Distress Tool, Social Determinants of Health, pain scale, diagnosis-specific scales, measures administered via third-party platforms (e.g. Press Ganey, Vizient), and internally developed tools

^g^Practices were only asked to respond to this item if they had mental health services available for oncology patients

^h^Includes neurocognitive and psychological evaluations, oncology specific caregiver support center, caregiver support, psychiatric medication management with psychiatrist, and no specification provided

### Availability of mental health care

Among NCORP practices that treat AYAs, 76% (*n* = 76) reported that mental health services were available onsite, while 23% (*n* = 23) reported that they did not have mental health services available onsite but had referral relationships with mental health providers in the community. One practice indicated that they did not have mental health services available on site or established referral relationships. Notably, the proportion of practices with a Pediatric Oncology Program that had onsite mental health services available was 89%, compared to 62% among practices without a Pediatric Oncology Program (*p* = 0.005). Among the 42 AYA-treating practices that reported having a dedicated Pediatric and/or AYA Oncology Program, 25 (60%) reported having a psychologist, 37 (88%) reported having a Social Worker, and 32 (76%) reported having a Child Life Specialist.

Among the 91 practices that reported routinely screening for distress, 76 (84%) reported that a primary strategy for managing oncology patients who screened positive for distress included referral to an on-site service (e.g., counselor, mental health professional). Assessment and management by an oncology provider (*n* = 62, 68%) and referral to an outside mental health professional (*n* = 53, 58%) were also common strategies. Furthermore, 62 (85%) practices with mental health services available “on site” could also deliver mental health services via telemedicine. Among practices with onsite mental health services, all but one reported the provision of individual psychotherapy or behavioral therapy (*n* = 75; 99%). Group psychosocial services were also commonly made available to patients (*n* = 60; 79%). These and related findings are presented in Table 2 and Supplemental Table 2.

### Differences in the availability of mental health care by practice group characteristics

Univariable logistic regression indicated that NCORP minority/underserved classification, COG affiliation, and proportion of Medicaid or uninsured patients were not associated with mental health service availability (see Tables 3, 4, and 5 for full results). Practices with AYA Oncology Programs were more likely to report onsite mental health services than those without AYA Oncology Programs (95% vs. 71.3%, Odds Ratio (OR) = 7.67, 95% Confidence Interval (CI) = 0.97–61, *p* = 0.054). Finally, practices serving greater than 100 new AYA cases per year and practices with Pediatric Oncology Programs were significantly more likely to have onsite mental health services (OR = 3.38, 95% CI = 1.19–9.54, *p* = 0.022 and OR = 3.64, 95% CI = 1.13–11.7, *p* = 0.030, respectively).

**Table 3:** Univariable models predicting the associations between NCORP practice characteristics and onsite availability of mental health services (MHS) among NCORP practices that treat AYAs (*N* = 100)

Practice characteristic	Description	MHS not available onsite (*n* = 24)	MHS available onsite (*n* = 76)	OR (95% CI)	*p*
NCORP Practice Classification	Minority/Underserved practice	6	19	Ref	0.99
	Non-Minority/Underserved practice	18	57	1.00 (0.35, 2.89)	
COG Practice	No	21	55	Ref	0.14
	Yes	3	21	2.67 (0.72, 9.90)	
Insurance: Medicaid	Percentage > national average (30%)	4	13	Ref	0.96
	Percentage ≤ national average (30%)	20	63	0.97 (0.28, 3.31)	
Insurance: Uninsured	Percentage > national average (10%)	3	9	Ref	0.93
	Percentage ≤ national average (10%)	21	67	1.06 (0.26, 4.29)	
AYA Population	< 100 new oncology cases ages 15–39/year	18	32	Ref	**0.022**
	≥ 100 new oncology cases ages 15–39/year	6	36	3.38 (1.19, 9.54)	
AYA Oncology Program	No	23	57	Ref	**0.054**
	Yes	1	19	7.67 (0.97, 61.0)	
Pediatric Oncology Program	No	20	44	Ref	**0.030**
	Yes	4	32	3.64 (1.13, 11.7)	

Bold indicates statistically significant *p*-values

**Table 4:** Univariable models predicting the associations between NCORP practice characteristics and onsite availability of mental health services (MHS) among NCORP practices that treat AYAs and have a Pediatric Oncology Program (*N* = 36)

Practice characteristic	Description	MHS not available onsite (*n* = 4)	MHS available onsite (*n *= 32)	OR (95% CI)	*p*
NCORP Practice Classification	Minority/Underserved practice	0	10	Cannot estimate	0.56
	Community practice	4	22		
COG Practice	No	1	12	Ref	0.63
	Yes	3	20	0.56 (0.05, 5.96)	
Insurance: Medicaid	Percentage > national average (30%)	2	9	Ref	0.38
	Percentage ≤ national average (30%)	2	23	2.56 (0.31, 21.0)	
Insurance: Uninsured	Percentage > national average (10%)	1	4	Ref	0.51
	Percentage ≤ national average (10%)	3	28	2.33 (0.19, 28.3)	
AYA Population	< 100 new oncology cases ages 15–39/year	4	7	Cannot estimate	**0.010**
	≥ 100 new oncology cases ages 15–39/year	0	20		
AYA Oncology Program	No	3	18	Ref	0.48
	Yes	1	14	2.33 (0.22, 24.9)	

Bold indicates statistically significant *p*-values

**Table 5:** Univariable models predicting the associations between NCORP practice characteristics and onsite availability of mental health services (MHS) among NCORP practices that treat AYAs and do not have a Pediatric Oncology Program (*N* = 64)

Practice characteristic	Description	MHS not available onsite (*n* = 20)	MHS available onsite (*n* = 44)	OR (95% CI)	*p*
NCORP Practice Classification	Minority/Underserved	6	9	Ref	0.41
	Non-Minority/Underserved	14	35	1.67 (0.50, 5.56)	
COG Practice	No	20	43	Cannot estimate	0.99
	Yes	0	1		
Insurance: Medicaid	Percentage > national average (30%)	2	4	Ref	0.91
	Percentage ≤ national average (30%)	18	40	1.11 (0.19, 6.63)	
Insurance: Uninsured	Percentage > national average (10%)	2	5	Ref	0.87
	Percentage ≤ national average (10%)	18	39	0.87 (0.15, 4.90)	
AYA Population	< 100 new oncology cases ages 15–39/year	14	25	Ref	0.49
	≥ 100 new oncology cases ages 15–39/year	6	16	1.49 (0.48, 4.69)	
AYA Oncology Program	No	20	39	Cannot estimate	0.31
	Yes	0	5		

Univariable models were also run separately for practices with and without a designated Pediatric Oncology Program. Among AYA-treating practices that reported having a Pediatric Oncology Program, sites that serve ≥ 100 new oncology cases ages 15–39 per year were more likely to have mental health services onsite (100% vs 63.6%, *p* = 0.010; see Table 4). Among AYA-treating practices that did not report having a Pediatric Oncology Program, no such association was observed (see Table 5).

A multivariable model that included these three AYA-relevant practice characteristics (practices serving at least 100 new AYA cases per year, practices with AYA Oncology Programs, and practices with Pediatric Oncology Programs) confirmed that practices seeing 100 or more new AYAs each year were more than three times as likely to have mental health services onsite than practices that see fewer AYAs (OR = 3.09, 95% CI = 1.04–9.18, *p* = 0.042). However, after adjusting for number of new AYA cases per year, no statistically significant differences were observed based on whether the practice had a formal Pediatric Oncology Program (*p* = 0.35) or AYA Oncology Program (*p* = 0.27; see Table 6).

**Table 6:** Multivariable models predicting the associations between AYA-relevant NCORP site characteristics and onsite availability of mental health services (MHS) among NCORP practices that treat AYAs (*N* = 100)

Practice characteristic	Description	OR (95% CI)	*p*
AYA Population	< 100 new oncology cases ages 15–39/year	Ref	**0.042**
	≥ 100 new oncology cases ages 15–39/year	3.09 (1.04, 9.18)	
AYA Oncology Program	No	Ref	0.27
	Yes	3.54 (0.37, 33.7)	
Pediatric Oncology Program	No	Ref	0.35
	Yes	1.87 (0.50–6.86)	

Variables that met a threshold value of *p* < 0.10 in univariable models were included in the multivariable models. Bold indicates statistically significant *p*-values

## Discussion

In this analysis of the 2022 Landscape Assessment of the NCORP, we found that the vast majority of practices report screening for distress, with nearly half using the NCCN Distress Thermometer [18]. However, there was heterogeneity in collection of other distress-related PROs. Further, nearly a quarter of practices that screened for distress reported not having mental health care services available to patients onsite to address reported distress. While the high prevalence of distress screening is encouraging, as it allows for identification of potential mental health needs in the cancer setting, our findings raise concerns about heterogeneity of measures used and interpretation of these measures in AYAs with cancer. Further, though many have on-site mental health services, over a quarter do not, which introduces room for additional barriers to accessing appropriate mental health care when elevated distress is determined.

The observed widespread use of distress screening is likely due to the mandate by the American College of Surgeon’s Commission on Cancer’s (ACS CoC) requirement for distress screening among adult cancer patients to maintain accreditation. This practice is further supported by recommendations from the American Society of Clinical Oncology [19] and the NCCN [2], as well as the Psychosocial Standards of Care for Children with Cancer and their Families [10] in the pediatric setting. However, The ACS CoC recommendations do not mandate which tool(s) should be used to evaluate distress [20]. In our sample, the NCCN Distress Thermometer [18] was the most widely used instrument. Although this measure is pragmatic for clinical practice and has been widely validated in the adult cancer population [8, 21, 22], research on its use in the AYA population remains limited [23]. The use of measures developed for AYAs would be ideal to ensure distress is accurately identified in this population. Indeed, the 2024 NCCN guidelines for AYA Oncology [24] suggest the use of the AYA Psycho-Oncology Screening Tool (AYA-POST), an age-specific tool developed in Australia to help clinicians improve psychosocial care for AYAs to ensure that areas of concern unique to this population are evaluated [25]. However, the use of an AYA-specific measure may not be practical in practices where patients are treated across a wide age range. In this case, it is important for practices to use measures validated with AYAs for the evaluation of distress, and to interpret findings in accordance with these validation studies. For example, a multinational validation of the distress thermometer with newly diagnosed AYAs indicated that a score of 5 yielded the best specificity and sensitivity [26], whereas a cut-off of 3 has been recommended for use based on findings from a large, heterogeneous sample of older cancer survivors [22]. The use of AYA-specific or AYA-validated tools ensures that clinically significant distress is accurately identified in this population [27, 28]. In the present study, data were not collected on the interpretation of distress screening tools. Future research is needed to better understand whether community oncology practices serving AYAs use different measures or interpretation strategies (e.g. cut-offs) to inform the care of AYA patients.

In addition to age-related concerns, our findings raise broader questions about the appropriateness and specificity of the questionnaires being used for cancer-related distress screening. For example, the PHQ measures [29, 30], Generalized Anxiety Disorder Scale-7 [31], and Hospital Anxiety and Depression Scale [32] were designed as screening tools to identify patients that may be experiencing clinical levels of depression and anxiety, rather than reporting generalized distress in the context of a life-threatening illness. As such, solely relying on these measures to evaluate distress in the context of cancer may result in under-identification of AYAs experiencing subclinical depression and anxiety symptoms or other types of distress symptoms but who could still benefit from psychosocial support in the context of cancer. The NCCN Distress Thermometer [18] consists of a single distress rating that ranges from 0 to 10 and a problem list of physical, emotional, social, practical, and spiritual or religious concerns, which helps to guide types of psychosocial referrals. Yet, the problem list has undergone limited validation and is not always administered alongside the distress thermometer rating [8]. In the present study, data was not collected on whether practices utilize the problem list in addition to the distress thermometer rating. However, we did find that the NCCN Distress Thermometer was the most used measure for evaluating both psychological and financial distress, suggesting that the use of the problem list is particularly important in assessing the type of distress and informing referral patterns [33]. For example, a patient experiencing heightened distress because of difficulties paying their medical bills may more directly benefit from a referral to a financial navigator rather than a mental health counselor. It is important to ensure that measures used for distress screening are both accurate and actionable. Measures developed specifically for this purpose such as the AYA-POST [25] discussed above (which has a concerns checklist that has undergone validation [26]) or the AYA Needs Assessment & Service Bridge (NA-SB) [34] may be particularly useful for ensuring that the psychosocial needs of AYAs are correctly identified and addressed by the appropriate team member. Indeed, the AYA-POST has been shown to facilitate prompt and appropriate referrals for this population [35]. Given that the 2022 Landscape Assessment was not specifically focused on AYAs, practices were not asked about the use of such age-specific measures. Future research is needed to better understand whether such tools are being used in AYA-treating community oncology practices, as well as the barriers and facilitators for doing so.

Finally, while distress screening was widely used in our sample, nearly a quarter of practices report not having any onsite mental health services available to AYAs who report distress. In practices without a dedicated Pediatric Oncology Program, this was true for nearly one-third of practices. Ultimately, the goal of distress screening is to facilitate referrals to psychosocial oncology specialists who can provide more extensive assessment and appropriate intervention [36]. Having onsite services is an important first step in ensuring this occurs. However, many health systems have long backlogs and delays in providing mental health services. Indeed, in the US, the median wait time for an in-person mental health appointment is 67 days and the median wait time for a telehealth appointment is 43 days [37]. While most practices reported the option for referral to an outside mental health provider, referrals to offsite services create additional barriers in access to and uptake of mental health care [38]. Such barriers have been demonstrated among U.S. adults with mental health challenges [39], and are likely even more problematic for AYAs, who have limited experience navigating the healthcare system and are already facing the significant challenges inherent in a cancer diagnosis [40]. Further, even when an AYA is able to access mental health care outside of the oncology setting, it is unlikely that such professionals are trained in providing care to cancer patients, and may not be able to fully address the unique needs of AYAs with cancer [41, 42].

One potential strategy to address lack of access to specialized mental health care for AYAs with cancer is the development of telehealth services offered by psychosocial oncology providers. Indeed, our study found that 62% of AYA-treating NCORP practices already offer virtual mental health services via telemedicine, suggesting this is a feasible approach. Notably, our study found that on-site mental health services were more likely to be available at sites that see at least 100 new AYAs annually. This suggests that there may be a critical mass of patients necessary to support such specialized services. This was particularly true at practices that did not report having a Pediatric Oncology Program, which tend to provide adolescent patients and their families with additional access to psychosocial care [43]. As such, one possibility may be that larger sites that employ mental health providers with expertise in working with AYAs could provide telehealth for patients seen at a region of community sites. Recent initiatives such as PSYPACT (an interstate compact designed to facilitate the practice of telepsychology across state boundaries) that allow for interjurisdictional practice have increased the feasibility of such strategies.

### Limitations

While this study makes important contributions to the literature on distress screening for AYAs, it is not without limitations. First, the findings from this study are specific to AYA-treating NCORP practices and may not be generalizable to other oncology settings. However, it is important to consider that NCORP practices represent a diverse array of oncology practices across the US, and thus these findings provide insight into real-world cancer care delivery in the community setting. Relatedly, data collected in the 2022 Landscape Survey are only available at the practice level. Thus, while only AYA-treating practices were included in this analysis, we could not determine whether AYA patients cared for at these practices consistently undergo distress screening, are evaluated with other PROs, and/or have access to onsite mental health care. Indeed, many NCORP sites provide pediatric and adult oncology care and may only provide distress screening and onsite mental health care in one or few of their clinical settings. As such, the reported rates of screening and mental health care availability are likely overestimates of the care available to AYAs. The 2022 Landscape Survey also lacked specificity; for example, while some practices reported using PROMIS measures, data on which measures were used were not collected. Furthermore, data from the Landscape Survey do not capture when or how distress screening is administered to AYAs or whether AYAs reporting distress accessed mental health services. Additional data (e.g., frequency of screening, pathways for referral to mental health care) are needed to better understand the effectiveness of distress screening in this setting at the patient level and develop strategies to improve psychosocial screening and service provision. Finally, nearly one-third of NCORP practices could not be categorized with regard to their AYA-treating status, due to missing information [15]. It is plausible that these practices may in fact treat AYAs and may have differential distress screening and access to onsite mental healthcare.

### Conclusions and future directions

The findings from this study provide two key insights: (1) while distress screening is widely implemented in AYA-treating NCORP practices, there is substantial variability in the screening tools used; (2) measures currently being used to evaluate distress at AYA-treating NCORP practices are not be specific or sensitive to the psychosocial concerns unique to AYAs. As such, our findings suggest that AYA-treating NCORP practices may need to modify existing distress screening procedures to enhance standardization across practices and ensure AYAs are evaluated appropriately. Ideally, screening should align with the recent core outcome set recommended for this population [44]. However, the need for these improvements should not deter practices from using widely applicable measures such as the NCCN Distress Thermometer if that is what is feasible; in doing so, however, it is important to apply appropriate cut points for the AYA population when more broadly applicable measures are used. Practices may also consider the use of Pediatric Distress Thermometer for AYA patients aged 21 or younger [45].

Finally, this study raises questions regarding the availability and accessibility of psycho-oncology care at the practice level for AYAs with cancer, particularly in practices that serve fewer AYAs and/or do not have a dedicated Pediatric Oncology Program. Ultimately, the goal of distress screening is timely referral for assessment and intervention; referral to off-site services may create barriers to accessing such care. Future studies should focus on understanding whether AYAs in the community setting who experience distress can and do access cancer-specific psychosocial care. Research should also focus on identifying modifiable barriers to care that will inform development of interventions that improve access to specialized psychosocial care for AYAs with cancer.

## Supplementary Information

Below is the link to the electronic supplementary material.

ESM 1 (DOCX 25.2 KB)

## Data Availability

Data can be requested from the NCORP Research Bases with approval from the Landscape Committee.
