# Supplementary material for: Distress screening and management for adolescents and young adults: 2022 NCI community oncology program landscape assessment
Source: Support Care Cancer. 2026 Jul 18;34(8):778. doi: 10.1007/s00520-026-11009-x (PMC13380545; doi:10.1007/s00520-026-11009-x)
Supplement: Supplementary file 1 — (DOCX 25.2 KB) [file 520_2026_11009_MOESM1_ESM.docx]

**Supplemental Table 1.** Landscape Assessment Measures

| **NCORP Practice Characteristics** |
| --- |
| ***Does your affiliate/subaffiliate include***  a. Outpatient oncology clinic(s) in or on a hospital campus  __ Yes How many different locations? ____  __ No  b. Free-standing outpatient oncology clinic(s) or private/group practice(s)  __ Yes How many different locations? _______  __ No  c. Inpatient services for oncology patients  __ Yes How many different locations? ____  __ No  d. Children’s hospital that treats pediatric oncology patients  __ Yes  __ No  ***Which of these best describes the ownership of your affiliate/subaffiliate?*** *(Please Select only one option)*  __ Independently owned (i.e. single hospital or small regional network [up to three hospitals) or an independent clinic/physician practice]  __ Hospital, clinic, or physician practice owned by a large regional/multi-state health system that does include a health plan  __ Hospital, clinic, or physician practice owned by a large regional/multi-state health system that does not include a health plan  __ HMO/Payer owned  __ Publicly owned (e.g. state, county, city)  __ University owned  __ Other, please specify: _________________________________  ***Does your affiliate/subaffiliate have a dedicated Pediatric Oncology Program, defined as tailored resources specifically targeting the pediatric population (which sometimes may include family caregivers) including treatment and supportive care?***  __ Yes  __ No  ***Which of the following services are provided on site at your affiliate/subaffiliate by your Pediatric Oncology Program?*** *(Select all that apply)*  __ Psychology  __ Social work  __ Financial navigation  __ Peer support  __ Chaplain  __Certified Child Life Specialist  __ Nurse or Social Work Navigator  __Lay navigator (a person who is not a nurse or social worker who provides navigation services; sometimes includes a trained person recruited from the community)  __ Provides post-treatment survivorship care for patients diagnosed as AYAs  __ Provides medical treatment and/or guidance regarding treatment  __ Other, specify: ______  ***Does your affiliate/subaffiliate have a dedicated Adolescent and Young Adult (AYA) Program, defined as tailored resources specifically targeting the AYA population including treatment and supportive care?***  __ Yes  __ No  ***Which of the following services are provided by your AYA Oncology Program?*** *(Select all that apply)*  __ Psychology  __ Social work  __ Financial navigation  __ Peer support  __ Chaplain  __ Certified Child Life Specialist  __ Nurse or Social Work Navigator  __ Lay navigator (a person who is not a nurse or social worker who provides navigation services; sometimes includes a trained person recruited from the community)  __ Provides medical treatment and/or guidance regarding treatment  __ Provides post-treatment survivorship care for patients diagnosed as AYAs  __ Work/ vocational rehabilitation  __Other, please specify: _______ |
| **Patient Reported Outcomes and Distress Screening** |
| ***Does your affiliate/subaffiliate routinely screen for distress (e.g., evaluate symptoms of anxiety & depression or psychosocial wellbeing in general) in your oncology patients?***  __ Yes  __ No   1. ***If Yes, What instrument(s) are used?*** *(Select all that apply):*   __ Generalized Anxiety Depression Scale (GAD-7)  __ Hospital Anxiety and Depression Scale (HADS)  __ Edmonton Symptom Assessment System (ESAS)  __ NCCN Distress Thermometer  __ Patient Health Questionnaire (PHQ)  __ Psychosocial Assessment Tool (PAT) (pediatric only)  __ Patient-Reported Outcomes Measurement Information System (PROMIS scale(s))  __ Other, please specify: _______________   1. ***If Yes, What is (are) the primary strategy (ies) used by your affiliate/subaffiliate to manage oncology patients who screen positive for anxiety or depression on the distress screening instrument?*** *(Select all that apply)*   __ Oncology provider assesses and manages patient  __ Referral to on-site service (e.g., counselor/mental health professional)  __ Referral to an outside counseling service/mental health professional  __ Referral to primary care provider  __ Other, please specify: _____________  __ N/A (No strategies are in place)  ***Does your affiliate/subaffiliate routinely use any other Patient Reported Outcomes (PROs) (e.g., FACT, SF-36, PROMIS) to inform clinical care (not exclusively as part of specific research protocols) for oncology patients?***  __ Yes  __ No   1. ***If Yes, are the following PRO tools routinely used clinically (i.e. not exclusively for research)?*** *(Select all that apply)*   __ FACT: Functional Assessment of Cancer Therapy  __ EORTC: European Organization for Research and Treatment of Cancer  __ SF-36: Short Form Survey  __ MDASI: MD Anderson Symptom Inventory  __ ESAS: Edmonton Symptom Assessment Scale  __ PROMIS: Patient Reported Outcomes Measurement Information System  __ Other single-item symptom severity rating (e.g., pain)  __ Other, please specify: ________  ***Are mental health services available for oncology patients at your affiliate/subaffiliate?***  __ Yes  __ No  __ No, we do not offer at our affiliate/subaffiliate but we have referral relationships with mental health providers in the community   1. ***If Yes, please specify what services are offered (Yes/No for each):***  \| **Service** \| \| **Yes** \| **No** \| \| --- \| --- \| --- \| --- \| \| Screening for mental health needs \|  \| \|  \| \| Individual psychosocial or behavioral therapy (e.g., coping support, counseling, smoking cessation, stress management) \|  \| \|  \| \| Couples and family therapy to address relationship issues, family issues and/or distress \|  \| \|  \| \| Group psychosocial services (e.g., support group, other psychosocial or psychoeducation group) \|  \| \|  \| \| Education classes around self-care for mental health (e.g., healthy behaviors, diet/nutrition, exercise, sleep, respite breaks) \|  \| \|  \| \| Help in getting respite care (e.g., when someone else takes care of the person a patient cares for, so that they can have a break) \|  \| \|  \| \| Other, please specify:_______ \|  \| \|  \|   ***b. If Yes, Are mental health services available via telemedicine for oncology patients at your affiliate/subaffiliate?***  __ Yes  __ No  __ No, but planning in progress |

**Supplemental Table 2.** Availability of mental health screening and services by practice group characteristics among NCORP practices that treat AYAs (N=100)

| **Practice Group Characteristics** | **Availability of Distress Screening** | | | | **Use of Other PROs** | | | | | **Availability of Mental Health Services Onsite** | | | | |  |
| --- | --- | --- | --- | --- | --- | --- | --- | --- | --- | --- | --- | --- | --- | --- | --- |
|  | **Yes**  **(n=91)** | | **No**  **(n=9)** | | | **Yes**  **(n=34)** | | **No**  **(n=66)** | | | **Yes**  **(n=76)** | | **No**  **(n=24)** | | |
| **NCORP Practice Affiliation** |  |  |  |  | |  |  |  |  | |  |  |  |  | |
| Non-Minority/Underserved (n=75) | 70 | (93.3%)^b^ | 5 | (6.7%) | | 26 | (34.7%) | 49 | (65.3%) | | 57 | (76.0%) | 18 | (24.0%) | |
| Minority/Underserved (n=25) | 21 | (84.0%) | 4 | (6.0%) | | 8 | (32.0%) | 17 | (68.0%) | | 19 | (76.0%) | 6 | (24.0%) | |
|  |  |  |  |  | |  |  |  |  | |  |  |  |  | |
| **Children’s Oncology Group Affiliation** |  |  |  |  | |  |  |  |  | |  |  |  |  | |
| Yes (n=24) | 21 | (87.5%) | 3 | (12.5%) | | 8 | (33.3%) | 16 | (66.7%) | | 19 | (79.2%) | 5 | (20.8%) | |
| No (n=76) | 70 | (92.1%) | 6 | (7.9%) | | 26 | (34.2%) | 50 | (65.8%) | | 57 | (75.0%) | 19 | (25.0%) | |
|  |  |  |  |  | |  |  |  |  | |  |  |  |  | |
| **NCORP sites serving Medicaid patients above the national average (30%)** |  |  |  |  | |  |  |  |  | |  |  |  |  | |
| Yes (n=17) | 11 | (12.1%) | 6 | (87.9%) | | 3 | (8.8%) | 14 | (91.2%) | | 13 | (76.5%) | 4 | (23.5%) | |
| No (n=83) | 80 | (87.9%) | 3 | (12.1%) | | 31 | (91.2%) | 52 | (8.8%) | | 63 | (75.9%) | 20 | (24.1%) | |
|  |  |  |  |  | |  |  |  |  | |  |  |  |  | |
| **NCORP sites serving Uninsured patients above the national average (10%)** |  |  |  |  | |  |  |  |  | |  |  |  |  | |
| Yes (n=12) | 12 | (13.2%) | 0 | (86.8%) | | 4 | (11.8%) | 8 | (88.2%) | | 9 | (75.0%) | 3 | (25.0%) | |
| No (n=88) | 79 | (86.8%) | 9 | (13.2%) | | 30 | (88.2%) | 58 | (11.8%) | | 67 | (76.1%) | 21 | (23.9%) | |
|  |  |  |  |  | |  |  |  |  | |  |  |  |  | |
| **Practice Settings^a^** |  |  |  |  | |  |  |  |  | |  |  |  |  | |
| Outpatient oncology clinics in or on a hospital  campus (n=84) | 77 | (91.7%) | 7 | (8.3%) | | 30 | (35.7%) | 54 | (64.3%) | | 66 | (78.6%) | 18 | (21.4%) | |
| Free-standing outpatient oncology clinics or  private/group practices (n=54) | 51 | (94.4%) | 3 | (5.6%) | | 18 | (33.3%) | 36 | (66.7%) | | 42 | (77.8%) | 12 | (22.2%) | |
| Inpatient services for oncology patients (n=81) | 74 | (91.4%) | 7 | (8.6%) | | 31 | (38.3%) | 50 | (61.7%) | | 64 | (79.0%) | 17 | (21.0%) | |
| Children’s hospital that treats pediatric oncology  patients (n=32) | 29 | (90.6%) | 3 | (9.4%) | | 10 | (31.3%) | 22 | (68.7%) | | 28 | (87.5%) | 4 | (12.5%) | |
| **Affiliate/sub-affiliate Ownership^a^** |  |  |  |  | |  |  |  |  | |  |  |  |  | |
| Independently owned (i.e., single hospital, small  regional network; n=18) | 16 | (88.9%) | 2 | (11.1%) | | 5 | (27.8%) | 13 | (72.2%) | | 11 | (61.1%) | 7 | (38.9%) | |
| Large regional multi-state health system that does  include a health plan (n=54) | 52 | (96.3%) | 2 | (3.7%) | | 19 | (35.2%) | 35 | (64.8%) | | 42 | (77.8%) | 12 | (22.2%) | |
| Large regional multi-state health system that does  NOT include a health plan (n=12) | 10 | (83.3%) | 2 | (16.7%) | | 3 | (25.0%) | 9 | (75.0%) | | 9 | (75.0%) | 3 | (25.0%) | |
| HMO/Payer owned (n=1) | 0 | (0%) | 1 | (100%) | | 1 | (100%) | 0 | (0%) | | 1 | (100%) | 0 | (0%) | |
| Publicly owned (n=8) | 6 | (75.0%) | 2 | (25.0%) | | 3 | (37.5%) | 5 | (62.5%) | | 7 | (87.5%) | 1 | (12.5%) | |
| University owned (n=4) | 4 | (100%) | 0 | (0%) | | 1 | (25.0%) | 3 | (75.0%) | | 4 | (100%) | 0 | (0%) | |
| Other (n=3) | 3 | (100%) | 0 | (0%) | | 2 | (66.7%) | 1 | (33.3%) | | 2 | (66.7%) | 1 | (33.3%) | |
| **AYA Population Served** |  |  |  |  | |  |  |  |  | |  |  |  |  | |
| <50 new cases^c^ ages 15-39/year (n=22) | 19 | (86.4%) | 3 | (13.6%) | | 4 | (18.2%) | 18 | (81.8%) | | 14 | (63.6%) | 8 | (36.4%) | |
| 50-99 new cases ages 15-39/year (n=28) | 26 | (92.9%) | 2 | (7.1%) | | 12 | (42.9%) | 16 | (57.1%) | | 18 | (64.3%) | 10 | (35.7%) | |
| 100-199 new cases ages 15-39/year (n=22) | 21 | (95.5%) | 1 | (4.5%) | | 7 | (31.8%) | 15 | (68.2%) | | 19 | (86.4%) | 3 | (13.6%) | |
| 200-499 new cases ages 15-39/year (n=14) | 13 | (92.9%) | 1 | (7.1%) | | 8 | (57.1%) | 6 | (42.9%) | | 12 | (85.7%) | 2 | (24.3%) | |
| >=500 new cases ages 15-39/year (n=6) | 6 | (100%) | 0 | (0%) | | 2 | (33.3%) | 4 | (66.7%) | | 5 | (83.3%) | 1 | (16.7%) | |
| # of new cases ages 15-39/year not reported (n=8) | 6 | (75.0%) | 2 | (25.0%) | | 1 | (12.5%) | 7 | (87.5%) | | 8 | (100%) | 0 | (0%) | |
|  |  |  |  |  | |  |  |  |  | |  |  |  |  | |
| **AYA Oncology Program** |  |  |  |  | |  |  |  |  | |  |  |  |  | |
| Yes (n=20) | 17 | (85.0%) | 3 | (15.0%) | | 5 | (25.0%) | 15 | (75.0%) | | 19 | (95.0%) | 1 | (5.0%) | |
| No (n=80) | 74 | (92.5%) | 6 | (6.5%) | | 29 | (36.3%) | 51 | (63.7%) | | 57 | (71.3%) | 23 | (28.7%) | |

^a^Practices were allowed to choose multiple options.

^b^Row percent.

^c^New oncology cases.
